# Preparation of l-Arginine Schiff Bases Modified Chitosan Derivatives and Their Antimicrobial and Antioxidant Properties

**DOI:** 10.3390/md20110688

**Published:** 2022-10-31

**Authors:** Jingmin Cui, Yan Sun, Linqing Wang, Qin Miao, Wenqiang Tan, Zhanyong Guo

**Affiliations:** 1 Key Laboratory of Coastal Biology and Bioresource Utilization, Yantai Institute of Coastal Zone Research, Chinese Academy of Sciences, Yantai 264003, China; 2 University of Chinese Academy of Sciences, Beijing 100049, China; 3 Center for Ocean Mega-Science, Chinese Academy of Sciences, Qingdao 266071, China; 4 School of Chemical and Materials Science, Ludong University, Yantai 264025, China

**Keywords:** chitosan, l-arginine Schiff bases, heterocyclic compounds

## Abstract

We successfully prepared a series of l-arginine Schiff bases acylated chitosan derivatives, aiming to improve the antioxidant activity and antimicrobial activity of chitosan by introducing a furan ring, pyridine ring, and l-arginine structure. The accuracy of the structures of ten compounds was characterized by FT-IR and ^1^H NMR. In terms of DPPH radical scavenging activity, except for compound CR3PCA, the scavenging rate of other compounds was higher than chitosan, especially CRCF and CRBF had strong scavenging abilities. At the same time, in the superoxide-radical scavenging activity assay, CRCF, CRBF, CR3PCA, CR2C3PCA, and CR2B3PCA were comparable to positive control at 1.60 mg/mL. Simultaneously, CRFF, CRCF, and CRBF had a certain inhibitory effect on *Botrytis cinerea*. Furthermore, the inhibitory effect of CRFF, CRCF, and CR3PCA on *Staphylococcus aureus* was very well, close to the positive control at 1.00 mg/mL. CRCF and CR2B3PCA showed better inhibitory effects on *Escherichia coli* than other compounds. The MTT assay was used to determine the cytotoxicity of the chitosan derivatives, which proved their safety to fibroblast cells. In summary, the study indicated that some of these compounds have the potential for further development and utilization in the preparation of antioxidants and antimicrobial agents.

## 1. Introduction

There is growing emphasis on the improvement of chitosan in recent years with a large number of synthetic chitosan derivatives demonstrating good biological activity [1,2,3,4]. Chitosan is a product of the deacetylation of chitin, which is widely found in nature and is the second largest natural polysaccharide on earth after cellulose [5]. Most of all, chitosan is the only cationic natural polysaccharide in nature, and it has certain antimicrobial and antioxidant activity in previous studies [2,6,7,8]. Furthermore, chitosan is non-toxic and harmless, which means that it has good biocompatibility and can be degraded by related enzymes after entering the human body [9]. Numerous researchers have demonstrated that chitosan derivatives have some antibacterial activity and antioxidant activity [10,11,12,13], including Amira A et al. who synthesized chitosan Schiff base derivatives containing different heterocyclic groups and determined their antimicrobial activity [14,15]. Cross-linked chitosan Schiff bases mixed with carboxymethyl chitosan (CMC) and graphene quantum dots (GQDs), might have the potential to be utilized for the treatment of *Helicobacter pylori* [16]. Methyl acrylate chitosan bearing *p*-nitrobenzaldehyde showed good capability at inhibiting the multidrug resistant (MDR) bacterial pathogens of skin burn infection [17]. Tan et al. synthesized *α*-lipoic acid grafted chitosan derivatives that could significantly improve the antioxidant activities, including superoxide anion, hydroxyl, and DPPH radicals scavenging activities, and reducing power [18]. Therefore, it is crucial to develop chitosan derivatives with high antimicrobial activity, good antioxidant activity, and low cytotoxicity.

l-arginine, as a precursor of many bioactive substances, can participate in regulating biological processes, and the metabolic pathways of l-arginine in vitro are as follows: l-arginine is decomposed into ornithine and urea under the action of arginase and then decomposed into citrulline and NO under the action of nitric oxide synthase [19]. At the same time, compounds containing guanidine have been widely studied and applied in pharmaceutical chemistry. The guanidine in l-arginine is protonated with a positive charge and interacts electrically with negatively charged bacterial cell surfaces to produce bacteriostatic effects [20]. In addition, studies have shown that l-arginine could induce disease resistance [21], attenuate oxidative stress, and inhibit the formation of atherosclerosis in hypercholesterolemia [22], etc.

There have been a series of studies on l-arginine-modified chitosan derivatives: Chitosan-*N*-arginine with different degrees of substitution was prepared and proved to have obvious antibacterial activity [23]. Chitosan-*N*-arginine/Hydroxypropyl methylcellose (HPMC) composite film was prepared by using HPMC as gelling agent and glycerin as a plasticizer, which had significant antibacterial activity and chitosan-*N*-arginine had no cytotoxicity to L929 cells [24]. 6-deoxy-6-arginine-modified chitosan not only increases the positive charge of the derivative with the guanidine of arginine but also retains the amino group after deprotection, showing a larger antibacterial circle than chitosan in antibacterial experiments [25]. Most studies focus on the modification of chitosan amino groups, such as forming amide bonds with the carboxyl groups of amino acids, respectively, or generating Schiff bases with the aldehyde groups of aldehydes [26,27,28]. However, few studies have connected amino acid Schiff bases to the chitosan framework.

Schiff bases are the product of the condensation of aldehydes/ketones and primary amines, and according to the different substituents, they can be divided into amines, ketones, semicarbazides, quinolines, azocyclics, guanidines, hydrazones, amino acids and so on [29,30]. The existence of the C=N double bond makes Schiff bases have a wide range of biological activities [31], and Schiff bases can form complexes with transition metals, lanthanides, actinides, and some main metallic elements, which have antimicrobial, antioxidant, anti-inflammatory, anticancer, and antitubercular activities [32,33]. So far, imine bonds were found in many drugs already approved for use, including furantoin, cimetidine, metformin, etc.

As usual, KOH is commonly added in the synthesis of amino acids Schiff bases, since most amino acids are insoluble in ethanol and methanol, adding strong bases can promote the dissolution of amino acids and the deprotonation of ligands [34,35]. However, the problem is that the synthetic Schiff bases are carboxylate, which is not conducive to the next step of the reaction to form amide bonds. The formation of Schiff bases between l-arginine and aldehydes has been studied extensively, for example: salicylaldehyde–arginine Schiff base, disalicylaldehyde–arginine Schiff base and metal–disalicylaldehyde–arginine Schiff base had high catalytic activity and remarkable selectivity under mild conditions [36]. Herein, a series of l-arginine Schiff bases acylated chitosan derivatives were reported: chitosan-l-arginine-furfural (CRFF), chitosan-l-arginine-5-methylfurfural (CRMF), chitosan-l-arginine-5-hydroxymethylfurfural (CRHMF), chitosan-l-arginine-5-chloro-furfural (CRCF), chitosan-l-arginine-5-bromo-furfural (CRBF), chitosan-l-arginine-2-pyridinecarboxaldehyde (CR2PCA), chitosan-l-arginine-3-pyridinecarboxaldehyde (CR3PCA), chitosan-l-arginine-4-pyridinecarboxaldehyde (CR4PCA), chitosan-l-arginine-2-chloro-3-pyridinecarboxaldehyde (CR2C3PCA), chitosan-l-arginine-2-bromo-3-pyridinecarboxaldehyde (CR2B3PCA), and their chemical structures were characterized by FT-IR and ^1^H NMR. The cytotoxicity was evaluated by MTT assay and the scavenging ability of superoxide radical and DPPH-radical were measured in this paper. Concurrently, two phytopathogenic fungi and two bacteria: *Fusarium graminearum* (*F. graminearum*), *Botrytis cinerea* (*B. cinerea*), *Staphylococcus aureus* (*S. aureus*), and *Escherichia coli* (*E. coli*) were selected to evaluate the antifungal property and antibacterial activity of the derivatives in vitro.

## 2. Results and Discussion

### 2.1. Chemical Synthesis and Characterization

#### 2.1.1. FT-IR Spectra

**Scheme 1:**
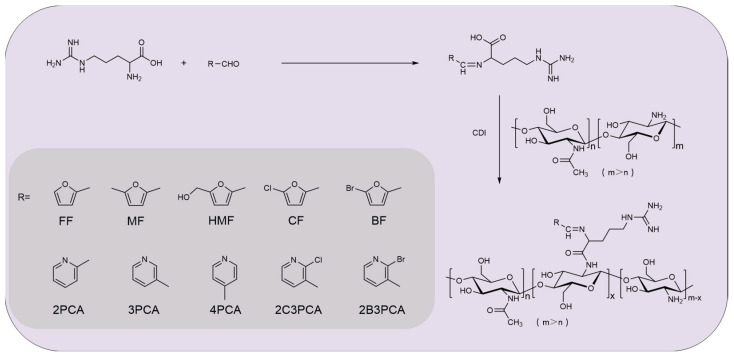
Synthesis pathway of chitosan derivatives.

Acylated chitosan derivatives with l-arginine Schiff bases were synthesized by a multi-step reaction (Scheme 1). The structures of chitosan derivatives were characterized by FT-IR and ^1^H NMR.

**Figure 1:**
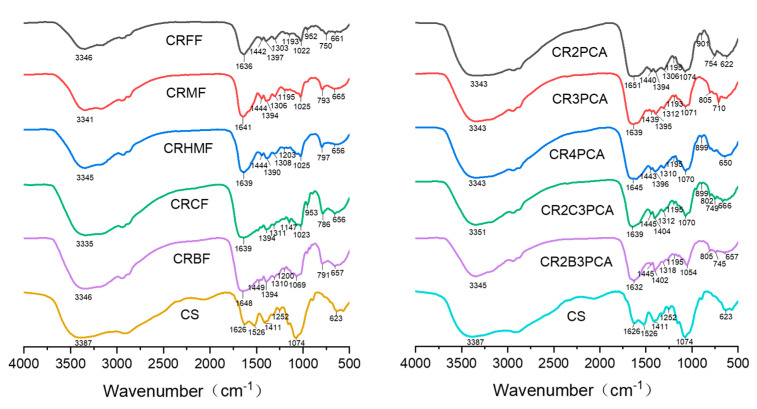
Fourier Transform Infrared (FT-IR) spectra of chitosan and chitosan derivatives.

First of all, as shown in Figure 1, we can see from the spectrum of raw material chitosan that the wide association peak near 3300 cm^−1^ includes O–H and N–H stretching absorption spectrum bands, the sway bending vibration of CH_2_ at 1411 cm^−1^, and the C–O stretching vibration of primary alcohol hydroxyl at 1074 cm^−1^. After the synthesis of ten compounds, it can be seen that the stretching vibration peak of NH_2_ disappears around 1526 cm^−1^, and a new wide peak appears around 1640 cm^−1^, which can be inferred to be the stretching vibration peak of C=N and C=O (amide bond). The stretching vibration peak at 1440 cm^−1^ is presumed to be CH_2_ bending vibration in l-arginine [37], the peak around 790 cm^−1^ is assumed to be the absorption peak of furan ring [38], and 750 cm^−1^ is attributed to the out-of-plane oscillations of C–H in the pyridine ring. In summary, it can be preliminarily proved that l-arginine Schiff bases combined successfully with chitosan.

#### 2.1.2. NMR Spectra

**Figure 2:**
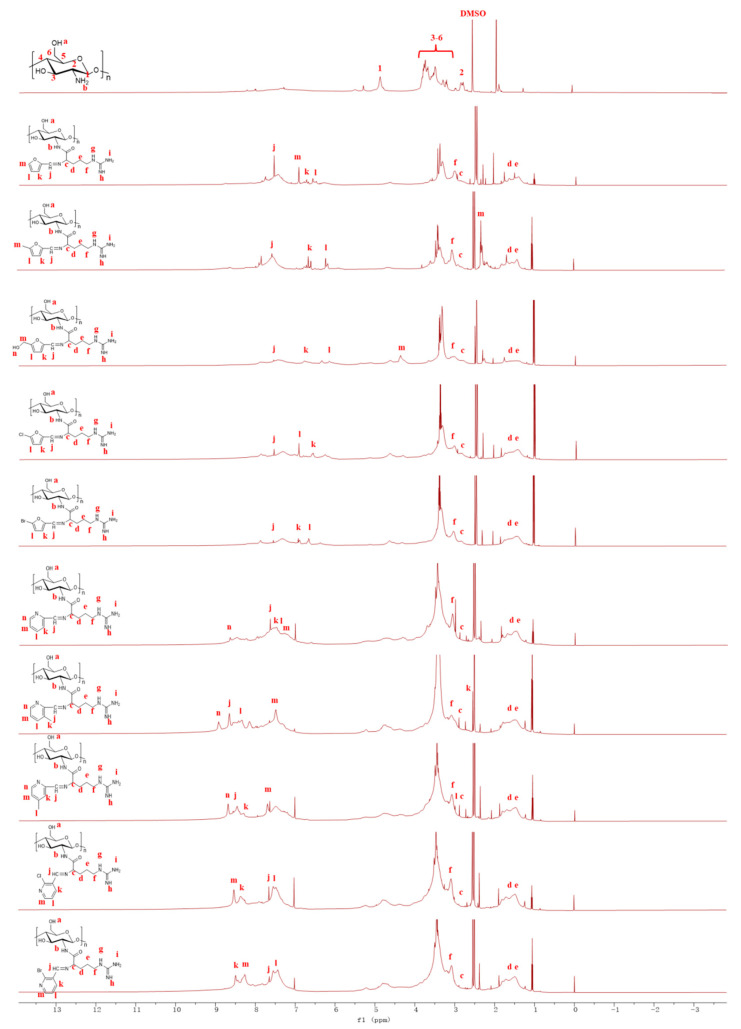
^1^H NMR spectra of chitosan and chitosan derivatives.

In order to compare the peak position of hydrogen atoms more directly, DMSO-d_6_ was selected as the solvent of chitosan and its derivatives. Usually, D_2_O is used as the solvent of chitosan in experiments, but the chitosan with molecular weight 8 kDa used in this experiment can be dissolved in DMSO-d_6_ under the condition of heating [39,40]. In Figure 2, The chemical shift of the hydrogen atom at the second position of chitosan is between 2.80 ppm and 3.00 ppm, and the characteristic peak of the hydrogen atom at the third to sixth positions of chitosan is between 3.20 ppm and 4.80 ppm [41]. After modification with l-arginine Schiff bases, the peak appears at 7.65 ppm, which is presumed to be the peak of hydrogen atoms on the C=N of Schiff bases. Additionally, the inactive hydrogen atom on l-arginine peaks at about 2.52 ppm and 1.50 ppm, respectively, which has also been mentioned in previous studies [42]. In addition, the active hydrogen peaks between 6.00–8.00 ppm on the furan ring and 7.00–9.00 ppm on the pyridine ring. These results can further prove the successful synthesis of chitosan derivatives modified with l-arginine Schiff bases.

#### 2.1.3. Yields and Degree of Substitution (DS) Analysis

The yields and DS of CRFF, CRMF, CRHMF, CRCF, CRBF, CR2PCA, CR3PCA, CR4PCA, CR2C3PCA and CR2B3PCA are shown in Table 1. Thus indirectly proved the successful synthesis of chitosan derivatives.

#### 2.1.4. Thermal Gravimetric Analysis (TGA) and Derivative Thermogravimetry (DTG)

**Figure 3:**
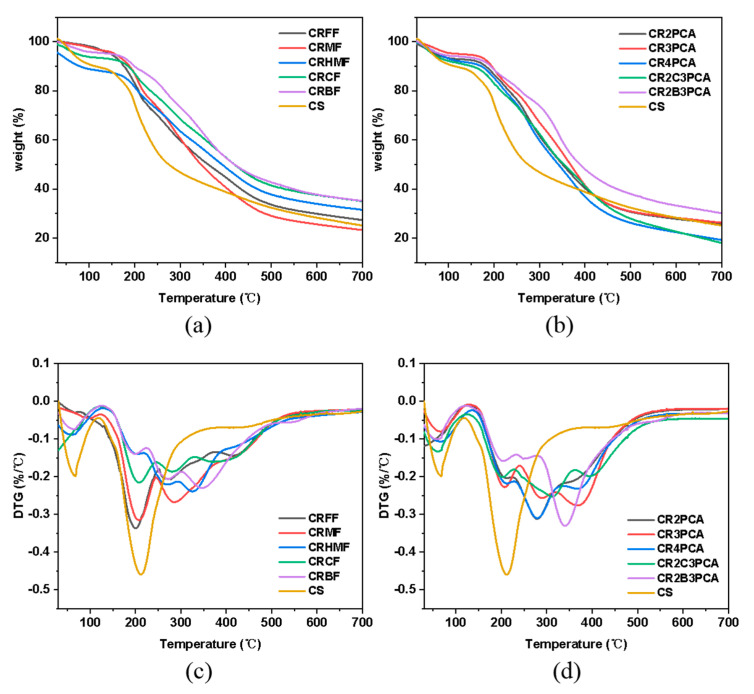
TGA (**a**,**b**) and DTG (**c**,**d**) curves of chitosan and chitosan derivatives.

Water-absorption and degradation behavior of chitosan and chitosan derivatives can be evaluated by TGA. In Figure 3, chitosan has two stages of weight loss, the first stage is around 30–120 °C with a loss of about 11% by weight for the evaporation of water molecules in chitosan; the second stage starts at 200 °C and reaches around 700 °C with a weight loss of about 74%, which is caused by the breakage and burning of the chitosan molecular chain [43,44]. In this case, chitosan reaches its maximum weight loss at 210 °C with a weight loss of 30.85%, which may be caused by the decomposition of the sugar rings at this point [45]. After modification, the chitosan derivatives have about four stages of weight loss, with a slower decomposition rate and higher thermal stability than chitosan. In the second stage, the maximum decomposition rates of the chitosan derivatives (CRFF, CRMF, CRHMF, CRCF, CRBF, CR2PCA, CR3PCA, CR4PCA, CR2C3PCA, CR2B3PCA) occurred at 198 °C, 207 °C, 199 °C, 210 °C, 201 °C, 207 °C, 206 °C, 208 °C, 203 °C, 201 °C, at which time the mass fractions were: 83.95%, 83.84%, 81.68%, 84.57%, 89.68%, 86.41%, 87.51%, 83.52%, 82.36%, 88.39%, and the weight losses were: 16.05%, 16.16%, 18.32%, 15.43%, 10.32%, 13.59% 12.49%, 16.48%, 17.64%, 11.61%.

### 2.2. Antioxidant Activity

**Figure 4:**
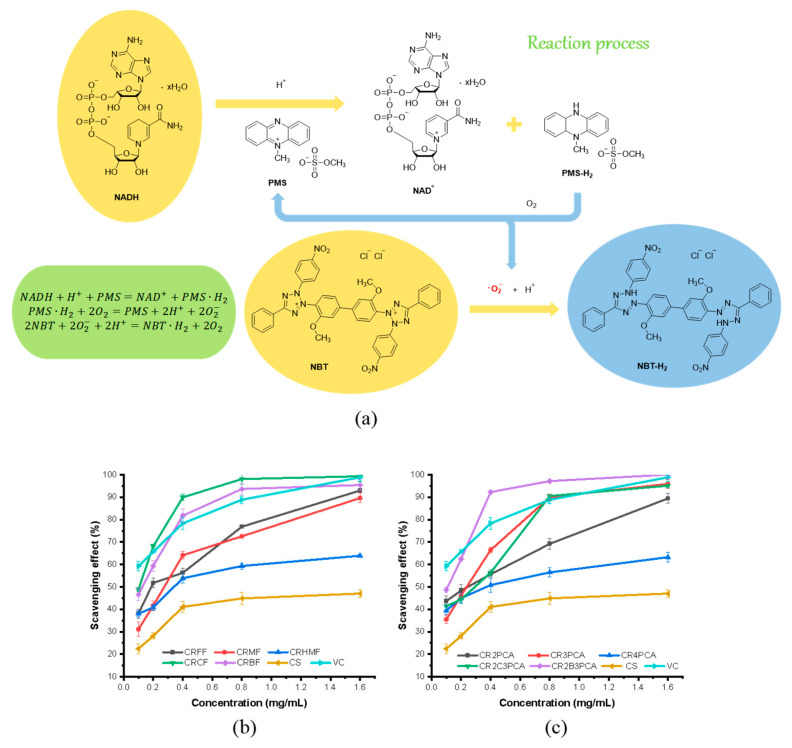
Reaction process of superoxide-radical scavenging activity assay (**a**), superoxide radical-scavenging capacity of chitosan and chitosan derivatives (**b**,**c**).

Superoxide anion radical ($O_{2}^{-}$·), as active intermediates in organism metabolism, is the product of single electron reduction of oxygen molecules. Abnormal levels of superoxide anion radicals in organisms are associated with a variety of diseases, such as diabetes, atherosclerosis, tumors, etc. The scavenging ability of ten newly synthesized compounds on superoxide anion free radical is shown in Figure 4, and the scavenging rate is higher than that of chitosan. According to the reaction process of the superoxide anion radical scavenging experiment, NAD^+^ and PMS-H_2_ are generated by the oxidation of NADH by PMS. PMS-H_2_ will generate superoxide anion radicals with oxygen in the air while NBT turns into NBT-H_2_, a blue compound when it encounters superoxide anion radicals. If the system contains antioxidants, the superoxide anion first associates with the antioxidants, and the production of NBT-H_2_ is reduced, leading to a decrease in the absorbance of the solution. We can infer that the ability of halogenated compounds to bind superoxide anion radicals is higher than that of other compounds, especially CR2B3PCA, CRCF, CRBF, and CR2C3PCA, which are comparable to Vitamin C (VC) at 1.60 mg/mL, the scavenging rates of superoxide anion radical were 100.00%, 99.37%, 95.95%, 95.45%, and 95.13%, respectively.

**Figure 5:**
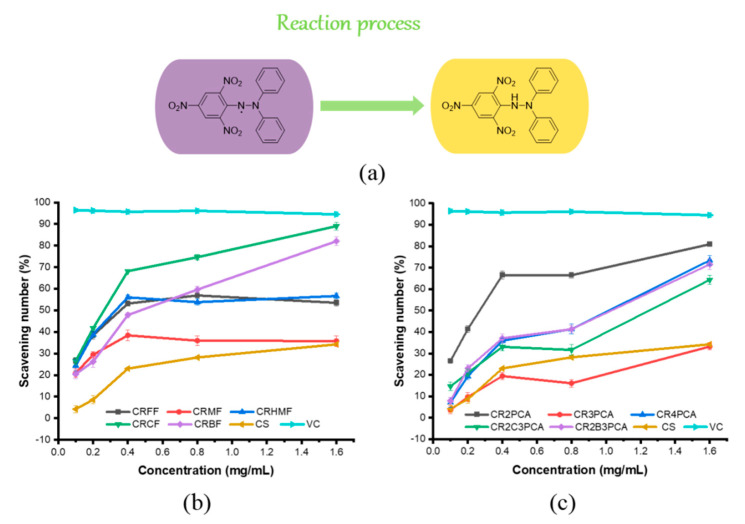
Reaction process of DPPH-radical scavenging ability assay (**a**), DPPH radical-scavenging capacity of chitosan and chitosan derivatives (**b**,**c**).

1,1-Diphenyl-2-picrylhydrazyl (DPPH) is a commonly used reagent to study the scavenging ability of free radicals, its structure is very stable due to electron delocalization. When DPPH is dissolved in ethanol, the solution appears purple and has a maximum absorption wavelength of 517 nm. The lone pair electron on the N atom connected with the trinitrobenzene ring can form a stable DPPH-H molecule when it meets the antioxidant, and the purple of the original solution will fade into yellow. Therefore, if the compound is a hydrogen donor, the absorbance of the solution will decrease, and the absorbance is linear with the concentration, thus reflecting its antioxidant activity. The scavenging ability of DPPH free radical was shown in Figure 5, the scavenging rate of other compounds was higher than that of chitosan except for compound CR3PCA. On the whole, CRCF, CRBF, and CR2PCA had strong scavenging abilities, and the scavenging ability of DPPH free radicals reached 89.01%, 82.04%, and 80.92% at the concentration of 1.60 mg/mL. In addition, CRCF, CRBF, CR4PCA, CR3PCA, and CR2C3PCA still showed a strong upward trend at the highest test concentrations, with obvious concentration dependence.

### 2.3. Antifungal Activity

*F. graminearum* is an epidemic plant fungus, which mainly occurs in the flowering stage and filling stage of the wheat growth period. If there is sufficient rain and high temperature, it is suitable for the growth of *F. graminearum* [46]. By the time wheat reaches maturity, it will have suffered a large loss of production if it is infected by *F. graminearum*. At the same time, it produces a variety of mycotoxins that can be toxic to humans and animals after use. *B. cinerea* has been widely studied as a model fungus, it can infect grapes, tomatoes, cucumbers, strawberries, and other crops, resulting in reduced production, so the design of more specific and effective fungicides is extremely important. There are two possible explanations for the inhibition mechanism of chitosan on fungi: one of them is that chitosan affects the activity of related enzymes in the cell wall, destroys the cell wall, and thus inhibits the growth of fungi, the other is that chitosan interacts with the cell membrane, increasing the permeability of the cell membrane and leaking the intracellular substances, leading to the death of fungi [2,47]. Studies have shown that introducing guanidine groups can enhance the ability of chitosan to inhibit fungi [48].

**Figure 6:**
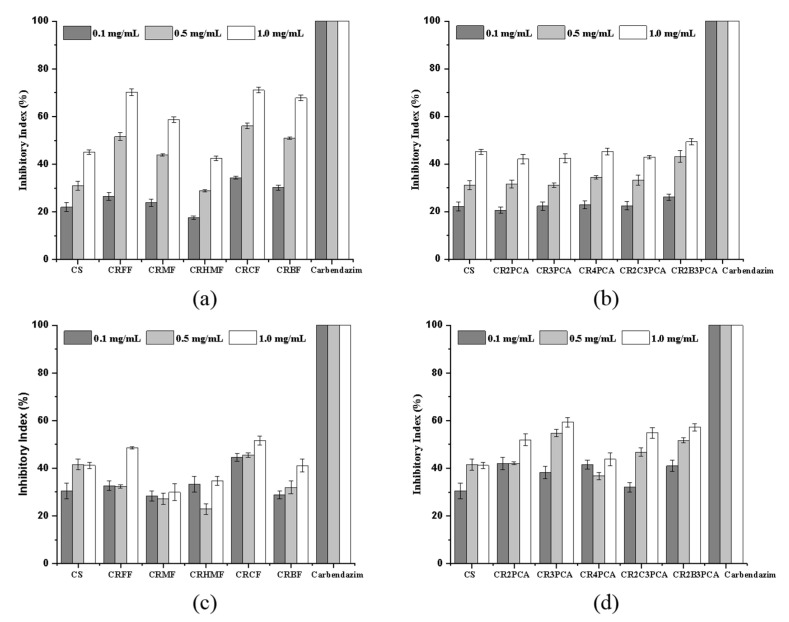
Antifungal activity of chitosan and chitosan derivatives against *B. cinerea* (**a**,**b**) and *F. graminearum* (**c**,**d**).

As shown in Figure 6a,b, all the synthesized compounds exhibit a concentration-dependent manner on *B. cinerea*, and the inhibitory effect of compounds containing furan rings was higher than that of compounds containing pyridine rings. Among them, the inhibition rates of CRFF, CRCF, and CRBF at 1.00 mg/mL were 70.22%, 71.07%, and 67.83%, respectively. Compared with chitosan, the antifungal activity of CRFF, CRCF, and CRBF was achieved at 32.66%, 33.51%, and 30.27% at the highest concentration tested, respectively. The five compounds containing pyridine ring showed little difference in their inhibitory activity against *B. cinerea*, and the inhibitory activity increased with the increase of concentration.

In Figure 6c,d, at the concentration of 1.00 mg/mL, the inhibition rate of CR3PCA was the highest (59.29%), followed by 57.15% of CR2B3PCA, 54.87% of CR2C3PCA and 51.99% of CR2PCA. In the series of compounds containing furan rings, at this concentration, the inhibition rate of CRCF was 51.63%, followed by that of CRFF was 48.58%. Even in the case of low concentration, at the concentration of 0.10 mg/mL, the inhibition rate was 44.50% for CRCF, 42% for CR2PCA, 41.50% for CR4PCA, and 41.03% for CR2B3PCA. At the same time, it can be seen that there is little difference between l-arginine Schiff bases containing furan rings and pyridine rings in the results of inhibition of *F. graminearum*. Similarly, halogens, hydroxyl groups, and methyl groups contained in heterocyclic Schiff bases have no significant effect on the inhibition effect.

### 2.4. Antibacterial Activity

**Figure 7:**
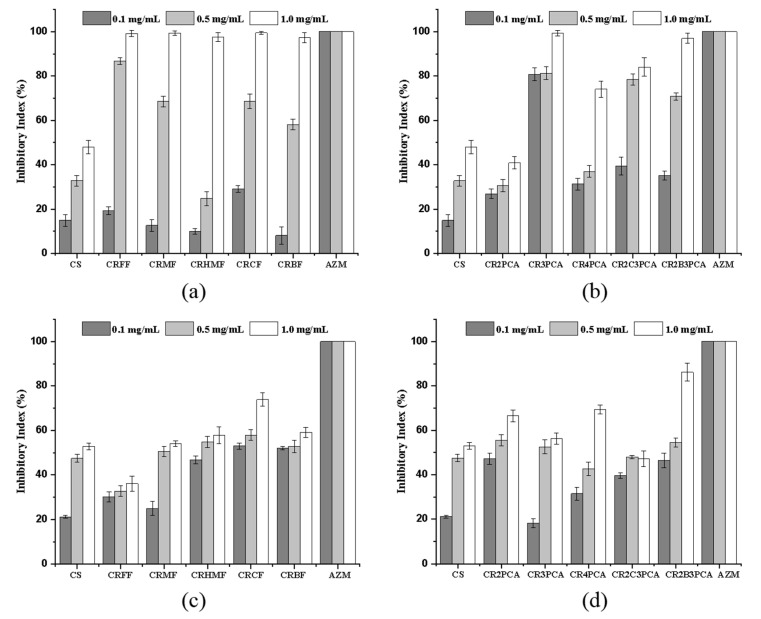
Antibacterial activity of chitosan and chitosan derivatives against *S. aureus* (**a**,**b**) and *E. coli* (**c**,**d**).

As shown in Figure 7, at the concentration of 1.00 mg/mL, the inhibition rates of CRFF, CRMF, CRHMF, CRCF, CRBF, CR3PCA, and CR2B3PCA against *S. aureus* were 99.17%, 99.35%, 97.56%, 99.47%, 97.30%, 99.31%, 96.98%, respectively. Gram-negative bacteria and Gram-positive bacteria have different cell wall compositions. Gram-positive bacteria (*S. aureus*) are composed of multilayer reticular peptidoglycan linked to teichoic acid and have a thicker cell wall. Peptidoglycan is a disaccharide unit containing a tetraceptide side chain, in which the amino acids in the tetraceptide vary by bacterial species. The teichoteic acid main chain is alternately linked by alcohol and phosphoric acid molecules, and the side chain is a single glucose or d-Ala. For Gram-negative bacteria (*E. coli*), the cell wall is thin and contains a single layer of peptidoglycan covered by an outer lipid membrane. From the experimental results, the sample solution at 0.10 mg/mL of gold glucose-inhibition ability is low, but at a high concentration (1.00 mg/mL) inhibition ability is very strong. Previous studies have speculated that chitosan derivatives form polymer membranes on cell surfaces that block the entry of nutrients [49]. For Gram-negative bacteria (*E. coli*), there is little difference between 0.50 mg/mL and 1.00 mg/mL in the inhibition ability of bacteria. It is speculated that the free positive charge and guanidine carried by chitosan can combine with the negatively charged components of the cell wall, resulting in the leakage of substances inside the cell holes and disturbing the normal physiological activities of bacteria [50].

### 2.5. Cytotoxicity Analysis

**Figure 8:**
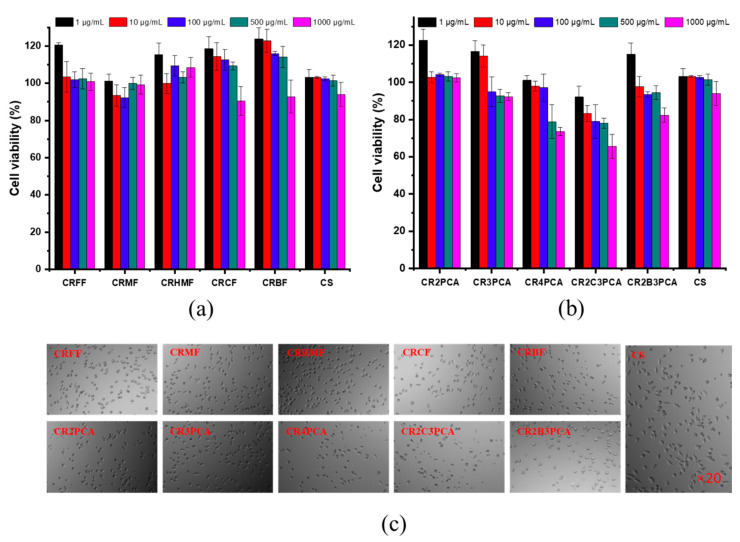
Cytotoxicity of chitosan and chitosan derivatives (**a**,**b**) and the sample concentration in the cell growth pictures (**c**) was 1000 μg/mL.

The cytotoxicity profile of chitosan and ten chitosan derivatives was evaluated in vitro by the MTT method, using L929 mice fibroblast cells line (L929, obtained from China Center for Type Culture Collection, Shandong Luye Pharmaceutical Co., Ltd., Yantai, China), which have a high differentiation capacity and ease of culture in vitro. The mechanism of MTT assay is that succinate dehydrogenase in the mitochondria of living cells restore exogenous MTT (3-(4,5-dimethyl-2-thizolyl)-2,5-diphenyltertazolium bromide) into formazan (a purple crystal insoluble in water). DMSO was added to dissolve formazan, and the number of cells was measured by measuring the absorbance of the solution. The cytotoxicity of the samples as shown in Figure 8a,b, it can be seen that CRFF, CRMF, CRHMF, CR2PCA, and CR3PCA were hardly toxic to L929 cells in the concentration range of 1–1000 µg/mL, while CRCF, CRBF, CR4PCA, CR2C3PCA, and CR2B3PCA had some cytotoxicity at 1000 µg/mL. For all samples, within the concentration range of the test sample, the cell viability exceeded 80%, which intuitively indicated low cytotoxicity. The morphology of the cells can be seen in Figure 8c. The normal cell morphology is spindle-shaped and adheres well to the cell wall, while the sample is toxic, the cells have a rounded morphology and no longer adhere to the cell wall. In conclusion, the non-toxicity of the chitosan derivatives was initially confirmed by the L929 cytotoxicity test and further experimental studies will be conducted if practical applications are considered.

## 3. Materials and Methods

### 3.1. Materials

Chitosan with a molecular weight of 8 kDa and 93% *N*-deacetylation was purchased from Golden-Shell Pharmaceutical Co., Ltd. (Zhejiang, China). l-Arginine and methanol were purchased from Sinopharm Chemical Reagent Co., Ltd., Shanghai, China. Furfural, 5-methylfurfural, 5-hydroxymethylfurfural, 5-chloro-furfural, 5-bromo-furfural, 2-pyridinecarboxaldehyde, 3-pyridinecarboxaldehyde, 4-pyridinecarboxaldehyde, 2-chloro-3-pyridinecarboxaldehyde, 2-bromo-3-pyridinecarboxaldehyde, 1,1′-carbonyldiimidazole (CDI) and dimethyl sulfoxide (DMSO) were purchased from Merck Life Science (Shanghai) Co., Ltd., Shanghai, China. Ethanol absolute was obtained from Fuyu Fine Chemicals Co., Ltd., Tianjin, China. Chloramphenicol and azithromycin were purchased from Aladdin Biochemical Technology Co., Ltd., Shanghai, China. Purified water was supplied by Wahaha Group Co., Ltd., Shandong, China.

### 3.2. Synthesis of Chitosan Derivatives

#### 3.2.1. Synthesis of l-Arginine Schiff Bases

l-arginine (1.74 g, 0.01 mol) was weighed and dispersed in 30 mL methanol at 60 °C for 2 h. The methanol solution with excess aldehydes was dropped into the above solution, heated to 70 °C, and reacted for 2 h after everything had dissolved. The solvent was removed by vacuum rotary evaporation, and excess aldehydes were removed by repeated washing and filtration with ethanol. The methods were slightly modified from those reported in the previous literature [36].

#### 3.2.2. Synthesis of Chitosan Derivatives

A total of 0.01 mol of l-arginine Schiff base was dissolved in 5 mL DMSO, and equal mole of CDI (1.62 g) was added to it for 12 h, then chitosan (0.005 mol, 0.8 g) was added, the reaction lasted for 24 h. After the reaction, ethanol was used for precipitation, washing, and lyophilization.

### 3.3. Analytical Methods

#### 3.3.1. Fourier Transform Infrared (FT-IR) Spectroscopy

The changes of functional groups of all chitosan derivatives were characterized by Nicolet IS 50 Fourier Transform Infrared Spectrometer (Thermo, Waltham, MA, USA), the test sample was scanned 16 times, and the infrared spectrum with a spectral range of 4000–400 cm^−1^ was obtained at a resolution of 4 cm^−1^. The dried samples were ground and pressed with KBr for testing at 20 °C.

#### 3.3.2. Nuclear Magnetic Resonance (NMR) Spectroscopy

The ^1^H NMR spectra of chitosan derivatives were recorded on Bruker AVIII 500 spectrometer (500MHz, Switzerland, provided by Bruker Tech. and Serv. Co., Ltd., Beijing, China), using DMSO as solvent. And the degrees of substitution (DS) of chitosan derivatives were calculated from the ^1^H NMR spectra.

#### 3.3.3. Degrees of Substitution (DS)

The calculation formula of DS was as follows: DS = H_a_/H_b_ (where H_a_ represents the integral area of a hydrogen atom in the C=N of the compound, H_b_ represents the integral area of the 1-position carbon bonded hydrogen proton on the main chain of chitosan.

#### 3.3.4. Thermal Stability

Thermal stability characterization of chitosan and its derivatives were characterized by Mettler 5 MP thermogravimetric analyzer (Mettler Toledo, Greifensee, Switzerland). The powdered samples were placed into a crucible and heated at 10 min^−1^ under a dynamic nitrogen atmosphere at a flow rate of 25 mL min^−1^ from 30 °C to 700 °C.

### 3.4. Antioxidant Assays

#### 3.4.1. Superoxide-Radical Scavenging Activity Assay

This experiment was measured according to the previous method [51]. The samples were freeze-dried to a constant weight and then added with deionized water to form a solution of 10 mg/mL, and then diluted with water into the solution to be tested with different concentrations: 0.20, 0.40, 0.80, 1.60, 3.20 mg/mL, respectively. Tris (Hydroxymethyl)aminoethane was configured with concentrated hydrochloric acid to form a Tris-HCl buffer (pH = 8.0). Concurrently, nicotinamide adenine dinucleotide reduced (NADH, 36.60 mg), nitro blue tetrazolium (NBT, 24.50 mg), and phenazine methosulfate (PMS, 1.80 mg) were weighed and dissolved in Tris-HCl buffer to a volume of 100 mL, respectively. For the experimental group, the solution was diluted to different concentrations and then 0.50 mL of NADH, 0.50 mL of NBT, and 0.50 mL of PMS were added. The blank group was replaced with deionized water, and the control group was replaced with Tris-HCl buffer instead of NADH solution, and then shaken well and reacted at room temperature for 5 min. After that, the absorbance of sample solutions with different concentrations at 560 nm was measured with a microplate reader. The formula of the superoxide-radical scavenging ability of chitosan derivatives is as follows:

(1) $$\mathrm{Scavenging}\;\mathrm{effect}\;\%=\left(1-\frac{A_{\mathrm{experimental}}-A_{\mathrm{control}}}{A_{\mathrm{blank}}}\right)\times 100$$

where A_experimental_ is the absorbance of the experimental group at 560 nm, A_control_ is the absorbance of the control group at 560 nm, and A_blank_ is the absorbance of the control group at 560 nm.

#### 3.4.2. DPPH-Radical Scavenging Ability Assay

Firstly, DPPH-ethanol solution was prepared: 35.50 mg DPPH was weighed and dissolved in anhydrous ethanol and transferred to a 500 mL volumetric bottle with anhydrous ethanol for constant volume. Then the sample solution was then prepared: the chitosan derivatives were freeze-dried to a constant weight and weigh 80 mg to prepare a sample mother solution of 10 mg/mL. The sample mother solution was diluted into the same volume (1.00 mL) but with different concentrations of 0.01 mg/mL, 0.02 mg/mL, 0.04 mg/mL, 0.08 mg/mL, and 0.16 mg/mL solutions. Then 2.00 mL DPPH-ethanol solution was added to the solution and reacted at room temperature for 20 min. After that, the absorbance of sample solutions with different concentrations at 517 nm was measured with a microplate reader. The blank group replaced the sample solution with the same volume of deionized water, and in the control group, DPPH ethanol was replaced with the same volume of anhydrous ethanol, excluding the interference of the sample solution’s own color. The formula of the DPPH radical scavenging ability for chitosan derivatives was as follows:

(2) $$\mathrm{Scavenging}\;\mathrm{effect}\;\%=\left(1-\frac{A_{\mathrm{experimental}}-A_{\mathrm{control}}}{A_{\mathrm{blank}}}\right)\times 100$$

where A_experimental_ is the absorbance of the experimental group at 517 nm, A_control_ is the absorbance of the control group at 517 nm, A_blank_ is the absorbance of the blank group at 517 nm. All experiments were repeated three times.

### 3.5. Antifungal Assays

The inhibition of chitosan derivatives on fungal pathogens was measured by mycelial growth rate method [52]. We selected two plant-pathogenic fungi: *F. graminearum* and *B. cinerea*, which have been selected as “The Top 10 fungal pathogens in molecular plant pathology” [53].

In this experiment, the mycelial growth rate method was used to test the inhibitory ability of chitosan derivatives on plant fungi, and the experimental methods were as follows: First, all compounds were dried to a constant weight, and then dissolved in sterile deionized water into a sample solution of 10 mg/mL. Solid media of different volumes were added to prepare the final concentration of 0.10 mg/mL, 0.50 mg/mL and 1.00 mg/mL. The positive control was carbendazim, and the blank control was sterile deionized water. After the frozen fungi were rejuvenated, the fungi were evenly distributed on the surface of solid medium by plate coating method, and cultured under the condition of temperature of 28 °C and humidity of 60% for 48 h. The 5 mm cake was taken with fungus cake puncher, inoculated in the center of solid medium containing different concentrations of compounds, and then cultured at the same humidity and temperature. Finally, the growth diameter of mycelia was measured by cross crossing method. The formula of fungal inhibition rates of chitosan derivatives was as follows:

(3) $$\mathrm{Fungistatic}\;\mathrm{Rate}\;\%=\left(1-\frac{D_{\mathrm{sample}}-5}{D_{\mathrm{blank}}-5}\right)\times 100$$

where D_sample_ is the diameter of sample medium and D_blank_ is the diameter of blank medium. All experiments were repeated three times.

### 3.6. Antibacterial Assay

Two kinds of model organisms were selected to test the antibacterial activity: *S. aureus* and *E. coli*. The plate colony counting method was used to measure the antibacterial activity of chitosan derivatives. The method is described in the previous experiment [54,55], the solid medium is mixed with the compound solution and finally prepared into 0.10 mg/mL, 0.50 mg/mL, and 1.00 mg/mL culture medium, in order to prepare the solid medium containing different concentrations of compounds. The pure colonies were selected and inoculated in a liquid medium after rejuvenating the frozen bacteria, and the bacterial solution was diluted after 4 h of growth. The concentration was measured as 10^5^ CFU/mL by absorbance, and 0.10 mL of the diluted liquid was absorbed and applied evenly on the surface of the solid medium containing the compound with a coating stick. The colony number was measured after growing at 37 °C for 12 h. The formula for bacteria inhibition rates of chitosan derivatives was as follows:

(4) $$\mathrm{Bacteriostasis}\;\mathrm{Rate}\;\%=\left(1-\frac{A_{\mathrm{sample}}}{A_{\mathrm{blank}}}\right)\times 100$$

where A_sample_ is the colony number of the sample to be tested. A_blank_ is the colony number of the blank sample. All experiments were repeated three times.

### 3.7. Cytotoxicity Assay

The cytotoxicity of chitosan and chitosan derivatives on L929 cells was determined by MTT assay at different concentrations of 1, 10, 100, 500, and 1000 μg/mL in vitro. L929 cells were incubated at 37 °C and 5% CO_2_. By cell recovery and passaging, L929 cells in the logarithmic growth phase (1 × 10^4^ cells/mL) were inoculated in 96-well plates. Cells were incubated to monolayer growth, then the supernatant was aspirated and discarded, followed by repeated washes with phosphate buffer solution (PBS). A series of concentrations of chitosan derivatives solution diluted in a culture medium was added into 96-well plates and incubated for 48 h. 20 µL of MTT solution and 80 µL of medium were added to each well, after 4 h of incubation, supernatants were removed, and 100 μL DMSO was added. The absorbance of each well at 490 nm was measured by a microplate reader (SuPerMax 3100, Flash, CN) and the cell viability was calculated according to the following formula:

(5) $$\mathrm{Cell}\;\mathrm{Viability}\;\%=\left(\frac{A_{\mathrm{sample}}-A_{\mathrm{blank}}}{A_{\mathrm{negative}}-A_{\mathrm{blank}}}\right)\times 100$$

where A_sample_ is the absorbance of the sample group at 490 nm, A_blank_ is the absorbance of the blank group at 490 nm, A_negative_ is the absorbance of the negative group at 490 nm. All experiments were repeated three times.

### 3.8. Statistical Analysis

The mean and standard deviation (SD) were calculated (*n* = 3) and one-way analysis of variance (ANOVA) and multiple comparison test (TUKEY) were used to analyze and compare the data. The results showed significant *p* < 0.05, indicating that the comparison between groups was statistically significant.

## 4. Conclusions

In this work, ten chitosan derivatives containing l-arginine Schiff base were prepared and their structural accuracy was verified by FT-IR and ^1^H NMR. Compared with chitosan, the antioxidant activities of the ten compounds were greatly improved, and they also had certain antifungal activities. Furthermore, these compounds also showed significant improvement in their ability to inhibit *S. aureus*. Schiff bases containing guanidine were grafted to chitosan by forming an amide group, which enriches the types of chitosan derivatives and expands the application range of chitosan derivatives as fungicides and antioxidants. It is hoped that this research will be further developed and utilized in agriculture or biomedical fields.

## Figures and Tables

**Table 1:** Yields and the DS of chitosan derivatives.

Compounds	Yields (%)	DS (%)	Compounds	Yields (%)	DS (%)
CS	-	-	CS	-	-
CRFF	87.69	65.36	CR2PCA	76.36	62.50
CRMF	83.60	63.69	CR3PCA	76.74	57.47
CRHMF	82.14	66.67	CR4PCA	80.68	64.51
CRCF	78.12	61.35	CR2C3PCA	78.38	53.47
CRBF	75.18	54.95	CR2B3PCA	87.88	63.29

## Data Availability

All data contained in the manuscript are available from the authors.
